# Supplementary material for: Spatial structure could explain the maintenance of alternative reproductive tactics in tree cricket males
Source: Biol Open. 2024 Jun 5;13(6):bio060307. doi: 10.1242/bio.060307 (PMC11179714; doi:10.1242/bio.060307)
Supplement: Supplementary information [file biolopen-13-060307-s1.pdf]

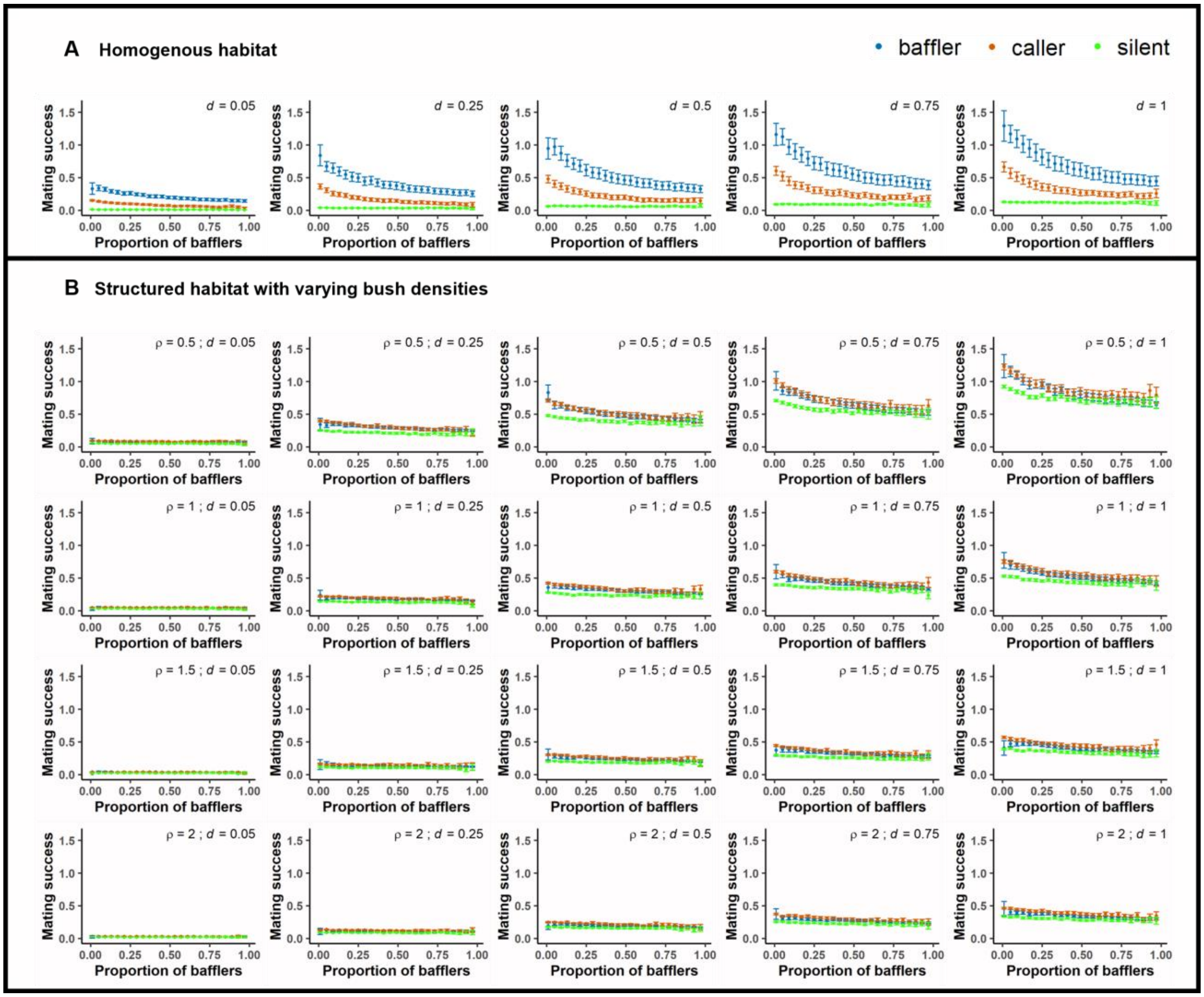

Fig. S1. Adding spatial structure not only weakens negative frequency dependence of baffling but also reduces disparity in mating success of the three ARTs. A) Mating success of the ARTs in the homogenous habitat at different population densities (i.e., individuals / m^2) given by *d*. B) Mating success of ARTs in structured habitats. Plots in each row correspond to a bush density (number of bushes / m^2) given by ρ and plots in each column correspond to a particular population density *d*. Error bars indicate 95% CIs. The downward slope of the mating success of bafflers is reduced in structured habitats (i.e., with bushes) compared to the homogenous habitat implying weakening of frequency dependence in structured habitats. Additionally, for any given population density, the slope of the mating success of bafflers reduces with increasing bush density (ρ). Finally, the disparity in mating success between baffling and the other ARTs (i.e., calling and silent) is reduced in structured habitats compared to homogenous habitats as indicated by overlapping data points for $\rho>0$.

Biology Open • Supplementary information

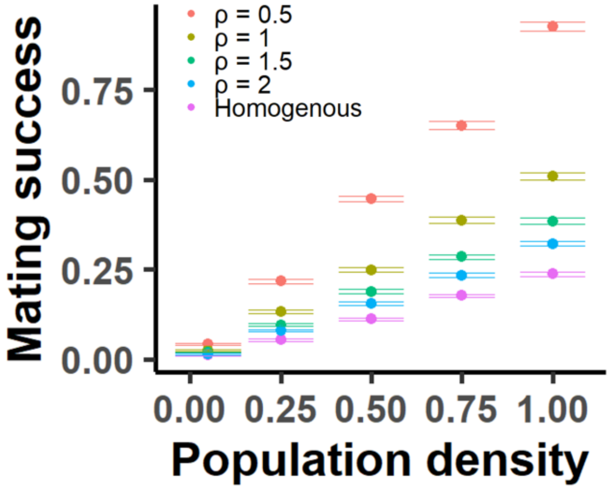

Fig. S2. Mating success of silent males in different habitats at different population densities (*d*) when only silent males and females were present. Error bars indicate 95% CIs.

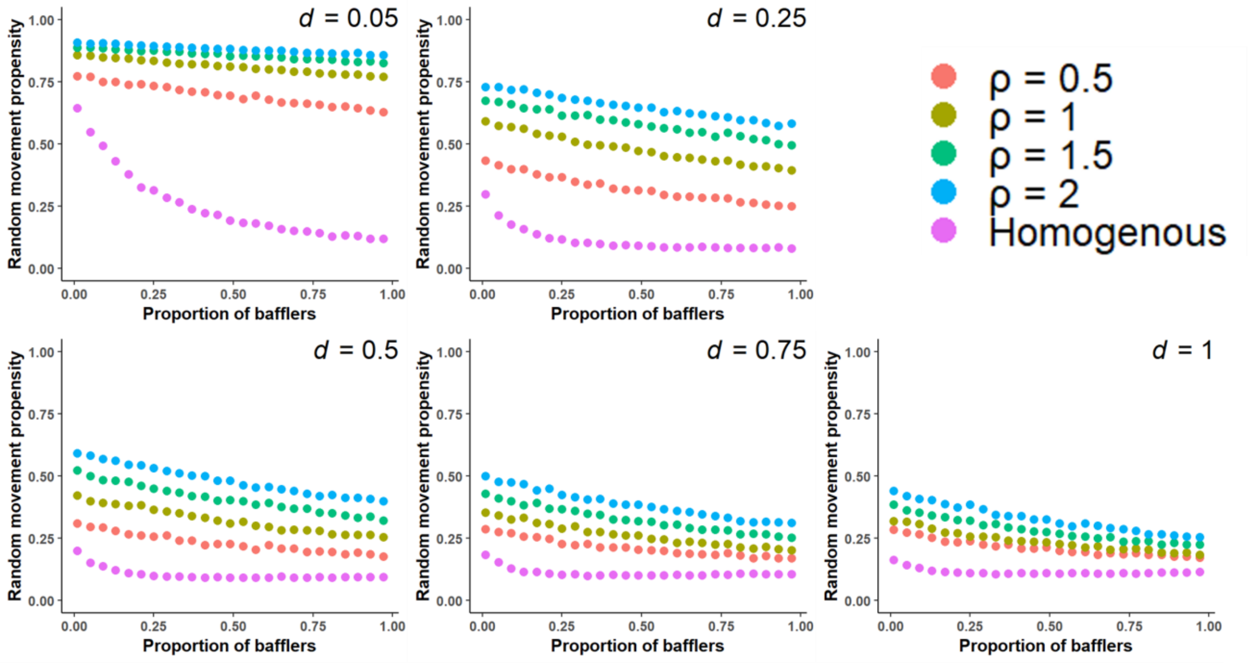

Fig. S3. Females show high propensity to move randomly in structured environments compared to homogenous environments. Random movement propensity:ratio of the mean number of timesteps in which a female performs random movement to the mean number of timesteps the female moves in a simulation run). The mean propensities were calculated after averaging over the mean number of timesteps (i.e., mean number of time steps a female moved randomly or moved at all) across 100 simulation runs.

Sensitivity analysis

The parameters used to construct the model are given in Table 1 (of the main manuscript). Here we show that our model was robust to some of these parameters. The list of parameters and their corresponding values for which sensitivity analyses were done are summarized in Table S1.

Table S1. Summary of parameters that were analysed for sensitivity. Our model was qualitatively robust to all specified values of the parameters.

Habitat type	Parameter	Values	Figure
Homogenous habitat; d = 1	male movement propensities (π_m)	0.35[#], 0.5, 0.9	S4
	female movement propensities (π_f)	0.2, 0.5, 0.8[#]	S5
	phonotaxis propensities (p) of mated females	0.35[#], 0.5, 1	S6
	caller : silent male ratio	1:1[#], 1:3, 3:1	S7
Structured habitats with ρ = 1; d = 0.5	male within-bush movement propensities (π_m)	0.35[#], 0.5, 0.9	S8
	female within-bush movement propensities (π_f)	0.2, 0.5, 0.8[#]	S9
	male across-bush movement propensities (π_m')	0.1197[#], 0.5, 0.9	S10
	female across-bush movement propensities (π_f')	0.1598[#], 0.4, 0.8	S11
	phonotaxis propensities (p) of mated females	0.35[#], 0.5, 1	S12
	caller : silent male ratio	1:1[#], 1:3, 3:1	S13

[#] indicates the value of the parameter used in Table 1.

Biology Open • Supplementary information

1. *Homogenous habitat*

In this section, we limited ourselves to the case of homogenous habitats with a population density d = 0.5. We varied some of the parameters (see below) and observed that the results of our model were qualitatively similar for a range of these parameter values. All other parameter values were identical to those mentioned in Table 1 unless otherwise mentioned.

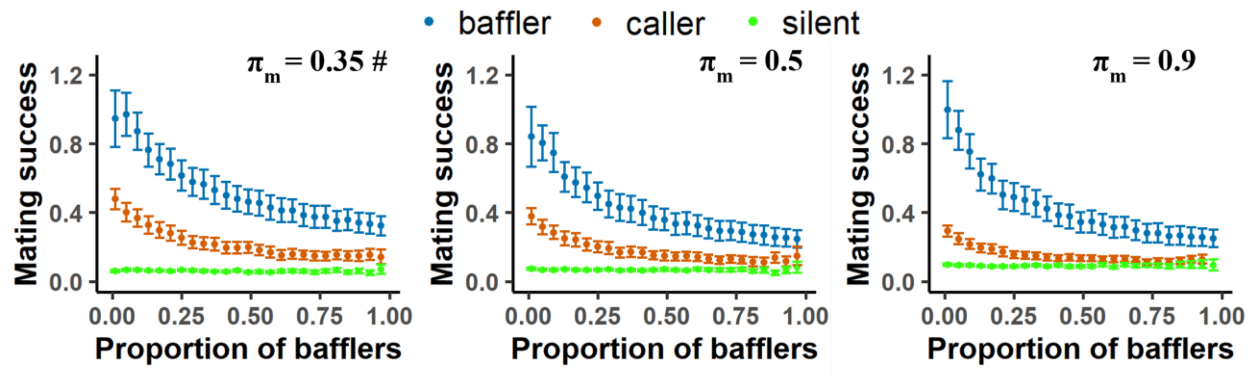

Fig. S4. Comparison of simulation results with homogenous habitats across different male movement propensities (π_m). # indicates that value of π_m is same as that in Table 1. Population density was kept constant at 0.5 individuals / m^2. Error bars indicate 95% CIs.

Biology Open • Supplementary information

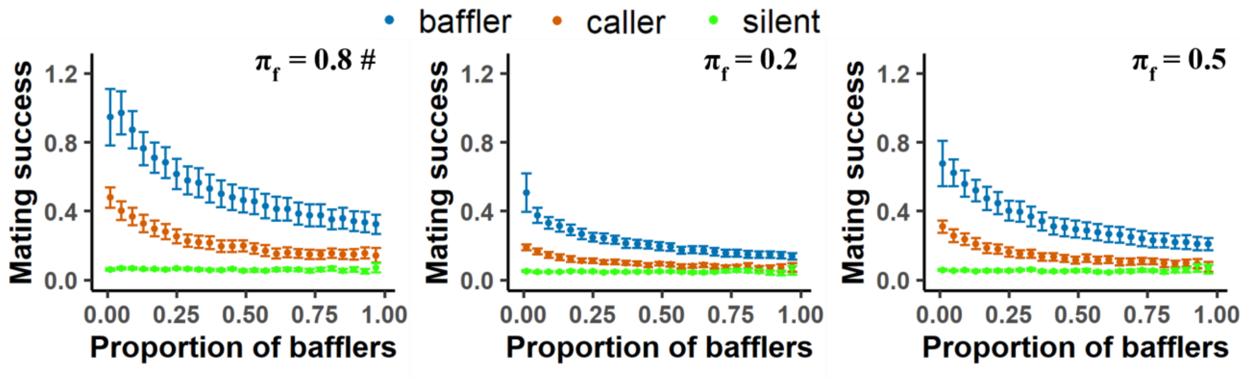

Fig. S5. Comparison of simulation results with homogenous habitats across different female movement propensities (π_f). # indicates that value of π_f is same as that in table S1. Population density was kept constant at 0.5 individuals / m^2. Error bars indicate 95% CIs.

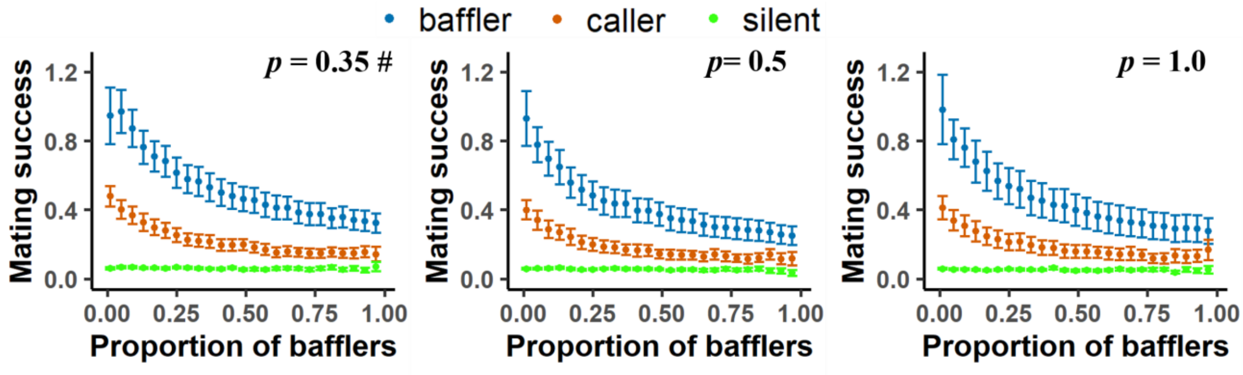

Fig. S6. Comparison of simulation results with homogenous habitats for different phonotaxis propensities (*p*) of mated females. # that indicates value of *p* is same as that in table S1. Population density was kept constant at 0.5 individuals / m^2. Error bars indicate 95% CIs.

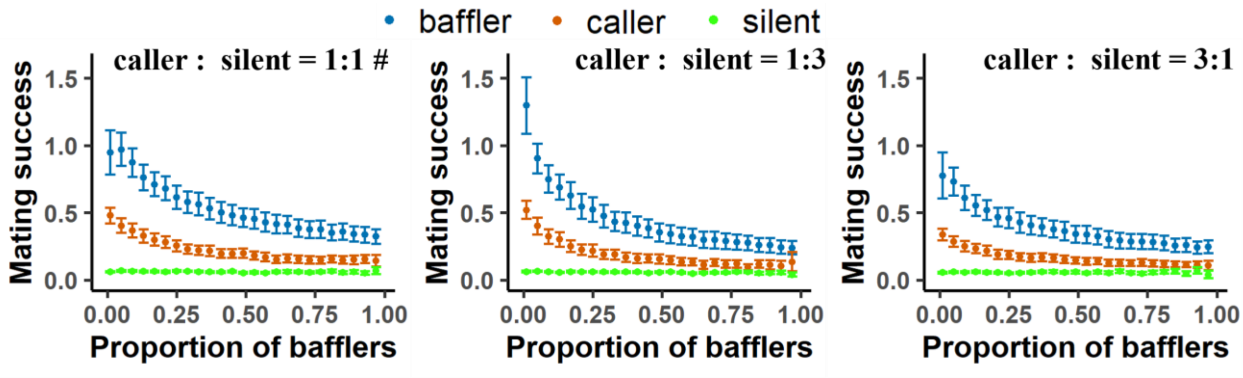

Fig. S7. Comparison of simulation results with homogenous habitats across different caller : silent ratios. # indicates that value of caller : silent ratio is same as that in Table 1. Population density was kept constant at 0.5 individuals / m^2. Error bars indicate 95% CIs.

2. *Structured habitat*

In this section, we limited ourselves to the case of structured habitats with a population density (d) equal to 0.5 individuals / m^2 and bush density (ρ) equal to 1 bushes / m^2. We varied some of the parameters (see below) and observed that the results of our model were qualitatively similar for a range of these parameter values. All other parameter values were identical to those mentioned in Table 1 unless otherwise mentioned.

Biology Open • Supplementary information

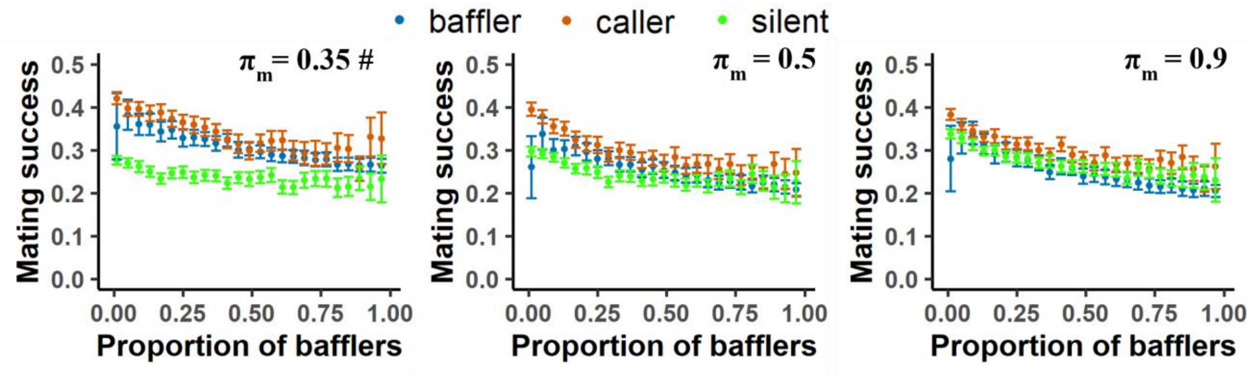

Fig. S8. Comparison of simulation results with structured habitats across different male within-bush movement propensities (π_m). # indicates that value of π_m is same as that in Table 1. Population density was kept constant at 0.5 individuals / m^2 and bush density was kept constant at 1 bush / m^2. Error bars indicate 95% CIs.

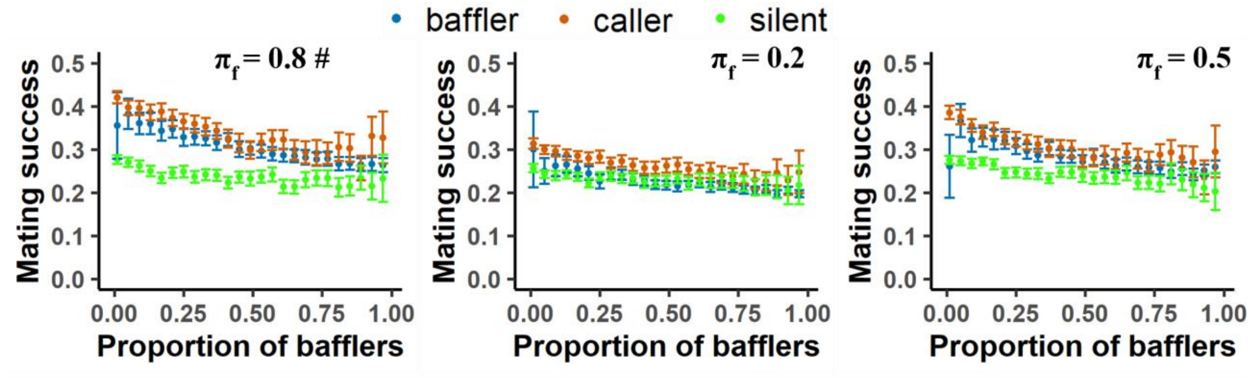

Fig. S9. Comparison of simulation results with structured habitats across different female within-bush movement propensities (π_f). # indicates that value of π_f is same as that in Table 1. Population density was kept constant at 0.5 individuals / m^2 and bush density was kept constant at 1 bush / m^2. Error bars indicate 95% CIs.

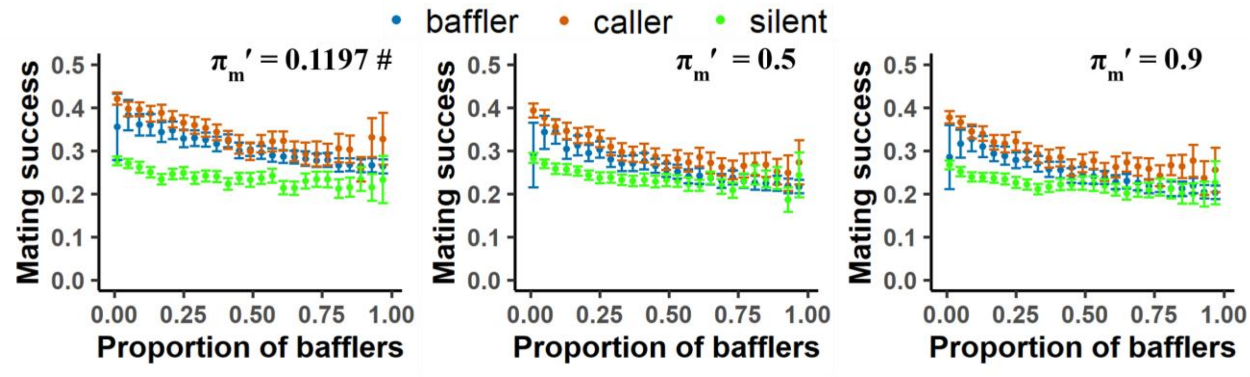

Fig. S10. Comparison of simulation results with structured habitats across different male across-bush movement propensities (π_m'). # indicates that value of π_m' is same as that in Table 1. Population density was kept constant at 0.5 individuals / m^2 and bush density was kept constant at 1 bush / m^2. Error bars indicate 95% CIs.

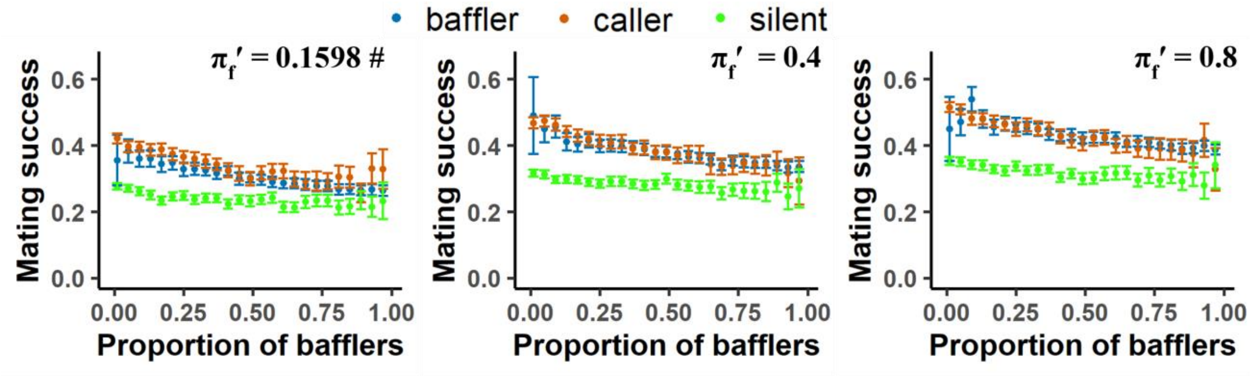

Fig. S11. Comparison of simulation results with structured habitats across different female across-bush movement propensities (π_f'). # indicates that value of π_f' is same as that in Table 1. Population density was kept constant at 0.5 individuals / m^2 and bush density was kept constant at 1 bush / m^2. Error bars indicate 95% CIs. Mating success of silent males tends to decrease relatively compared to that of callers and bafflers as across-bush movement propensity of females increases, thus, implying that signaling may be beneficial when females show reduced site fidelity. However, baffling is not more beneficial than calling even when across bush propensity of females is increased.

Biology Open • Supplementary information

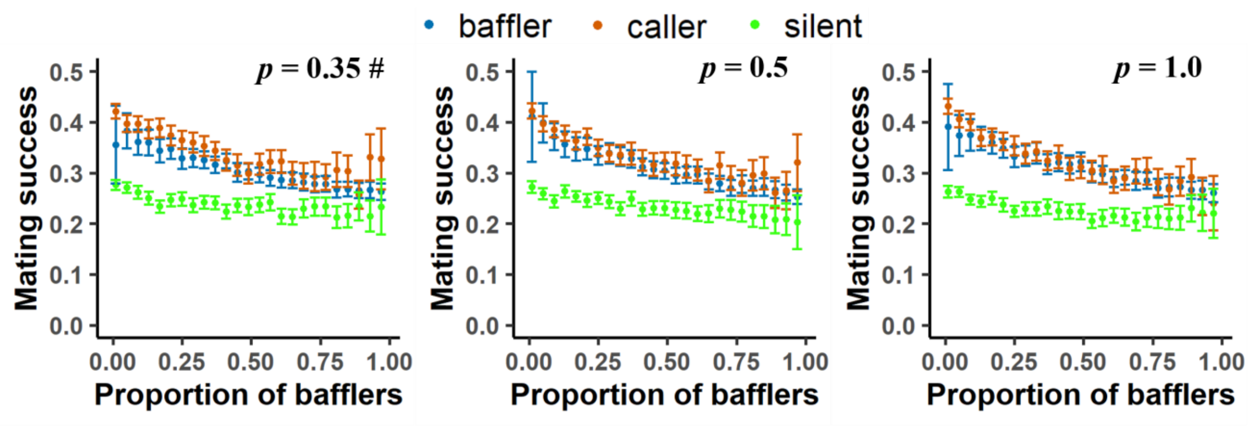

Fig. S12. Comparison of simulation results with structured habitats across different female mated phonotaxis propensities (*p*). # indicates that value of *p* is same as that in Table 1. Population density was kept constant at 0.5 individuals / m^2 and bush density was kept constant at 1 bush / m^2. Error bars indicate 95% CIs.

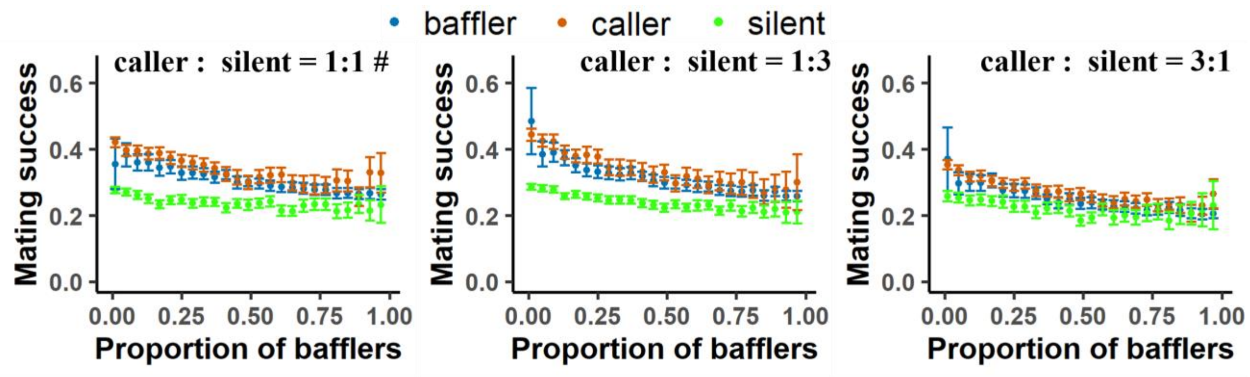

Fig. S13. Comparison of simulation results with structured habitats across different caller : silent ratios. # indicates that value of caller : silent males ratio is same as that in Table 11. Population density was kept constant at 0.5 individuals / m^2 and bush density was kept constant at 1 bush / m^2. Error bars indicate 95% CIs.

Biology Open • Supplementary information

References

Deb, Rittik, Modak, S., & Balakrishnan, R. (2020). Baffling: a condition-dependent alternative mate attraction strategy using self-made tools in tree crickets. *Proceedings of the Royal Society B: Biological Sciences, 287*(1941), 20202229. https://doi.org/10.1098/rspb.2020.2229

Modak, Sambita, Brown, W. D., & Balakrishnan, R. (2021). Decoupling of female phonotaxis and mating propensity in a tree cricket. *Behavioral Ecology and Sociobiology*, *75*(10). https://doi.org/10.1007/s00265-021-03084-3

Torsekar, V. R., & Balakrishnan, R. (2020). Sex differences in alternative reproductive tactics in response to predation risk in tree crickets. *Functional Ecology*, *287*(1941), 20202229. https://doi.org/10.1111/1365-2435.13652

Biology Open • Supplementary information
